# An online system for metabolic network analysis

**DOI:** 10.1093/database/bau091

**Published:** 2014-09-27

**Authors:** Abdullah Ercument Cicek, Xinjian Qi, Ali Cakmak, Stephen R. Johnson, Xu Han, Sami Alshalwi, Zehra Meral Ozsoyoglu, Gultekin Ozsoyoglu

**Affiliations:** ^1^Lane Center for Computational Biology, School of Computer Science, Carnegie Mellon University, Pittsburgh, PA 15222, USA, ^2^Electrical Engineering and Computer Science, Case Western Reserve University, Cleveland, OH 44106, USA and ^3^Department of Computer Science, Istanbul Sehir University, Istanbul 34662, Turkey

## Abstract

Metabolic networks have become one of the centers of attention in life sciences research with the advancements in the metabolomics field. A vast array of studies analyzes metabolites and their interrelations to seek explanations for various biological questions, and numerous genome-scale metabolic networks have been assembled to serve for this purpose. The increasing focus on this topic comes with the need for software systems that store, query, browse, analyze and visualize metabolic networks. PathCase Metabolomics Analysis Workbench (PathCaseMAW) is built, released and runs on a manually created generic mammalian metabolic network. The PathCaseMAW system provides a database-enabled framework and Web-based computational tools for browsing, querying, analyzing and visualizing stored metabolic networks. PathCaseMAW editor, with its user-friendly interface, can be used to create a new metabolic network and/or update an existing metabolic network. The network can also be created from an existing genome-scale reconstructed network using the PathCaseMAW SBML parser. The metabolic network can be accessed through a Web interface or an iPad application. For metabolomics analysis, steady-state metabolic network dynamics analysis (SMDA) algorithm is implemented and integrated with the system. SMDA tool is accessible through both the Web-based interface and the iPad application for metabolomics analysis based on a metabolic profile. PathCaseMAW is a comprehensive system with various data input and data access subsystems. It is easy to work with by design, and is a promising tool for metabolomics research and for educational purposes.

**Database URL**: http://nashua.case.edu/PathwaysMAW/Web

## Introduction

Metabolomics is a relatively new ‘omics’ platform in life sciences research. The advancements in analytical methodology and high-throughput rates have led to the collection of large metabolic data sets. Metabolic profiles and genome-scale metabolic networks (1) are used in various contexts, such as (i) predicting flux distribution for the metabolic activity over the network [metabolic control analysis (MCA) (2), flux balance analysis (FBA) (3) and constraint-based methods] and (ii) drug discovery and disease research (4–7). The increase in the number and importance of metabolic networks has come with the need for carefully designed databases to store/organize metabolic networks, and efficient online tools to browse/ analyze/visualize metabolic data.

The goal of PathCase^MAW^ (Metabolic Analysis Workbench) is to provide a metabolic network database and a Web- or tablet-based system that enables users to interact with the underlying metabolic network. PathCase^MAW^ provides the following functionalities:

1. A metabolic network database that captures the metabolic network with a compartment hierarchy and metabolic regulation relationships.
2. A Web site that (i) enables users to browse pathways, reactions, metabolites/metabolite pools and compartments stored in the database, (ii) provides several built-in queries and interactive visualization and (iii) has the integrated steady-state metabolic dynamics analysis (SMDA) tool. SMDA tool takes a set of metabolite measurements and a metabolic subnetwork stored in the PathCase^MAW^ database as input. Then, it produces all possible steady-state flow scenarios (called flow graphs) for the selected subnetwork as output (that are consistent with the observed metabolite measurements and the underlying biochemistry) (available at http://nashua.case.edu/PathwaysMAW/web/).
3. An iPad application that has all capabilities of the Web-based PathCase^MAW^ system with the exception of browsing/querying (available at Apple AppStore).
4. An offline metabolic network editor with visualization capabilities that enables users to create their own network in a user-friendly way.
5. An SBML Parser to parse and store genome-scale reconstructed metabolic networks [e.g. Recon 1 of humans (8)] into the PathCase^MAW^ database.

Currently, the PathCase^MAW^ system works on a manually created (and generic) mammalian metabolic network, which is obtained from the metabolic atlas by Selway *et al.* (9). We also have three genome-scale reconstructed networks hosted and available on the sister PathCase^RCMN^ (PathCase Reconstructed Metabolic Networks) Web site (10). Source codes of the Web interface, PathCase^MAW^ editor, SBML Parser, as well as the database schema are available on request for academic users to create their own networks and to host/access them. User-created networks can also be hosted on the PathCase^RCMN^ Web site on request.

## Implementation

In this section, we summarize the design and implementation details of the PathCase^MAW^ system.

### Architecture

PathCase^MAW^ has a two-tiered client-server software architecture, with a thick client. On the client side, there are four applications.

*PathCase^MAW^ Web interface*. Via any Web browser, users can access, visualize, query and analyze the data stored in the PathCase^MAW^ database. Application is written in C# language using .NET environment. IIS 7.0 is used as the Web server.

*PathCase^MAW^ iPad application*. Users can visualize pathways stored in the database, and use the SMDA tool. iPad application can be downloaded for free from the Apple App store, and runs on iPad2 and newer models. The application uses a buffer for pathway data in its flash memory for fast access, and to reduce data transfer from the server. The iPad application data are updated whenever the PathCase^MAW^ database is updated. The communication between the iPad application and the server PathCase^MAW^ database is handled through the Web services (XML and GML documents are used as the data exchange format). Users are able to use iPad’s multi-touch screen to visualize pathways and to run the SMDA tool in a mobile tablet environment.

*PathCase^MAW^ editor*. This application is used by data owners to create/update metabolic networks in the PathCase^MAW^ database. This is an offline tool and is implemented in JAVA to provide platform independence.

*PathCase^MAW^ SBML parser*. This is a console application that takes as input an SBML file for a metabolic network, and parses it into the PathCase^MAW^ database as a separate metabolic network. It is written in C# using .NET framework.

On the server side, we have the following components.

*PathCase^MAW^ database*. This application is stored in Microsoft SqlServer 2008 and is accessed through the Data Access Library.

*The data access library*. This contains (i) database wrappers that abstract the database schema, (ii) built-in queries and (iii) an XML generator that generates a metabolic network/subnetwork/pathway visualization file, which is then passed to either (a) JAVA applet of the PathCase^MAW^ client-side browser, (b) PathCase^MAW^ iPad application or (c) the PathCase^MAW^ editor.

*The web content UI manager*. This handles synchronous or asynchronous requests sent from the client browser, and responds in HTML format.

*The SMDA tool*. This tool implements the SMDA algorithm (11, 12).

*The Web services*. This component is used for data access during query processing and for network visualization.

**Figure 1.**
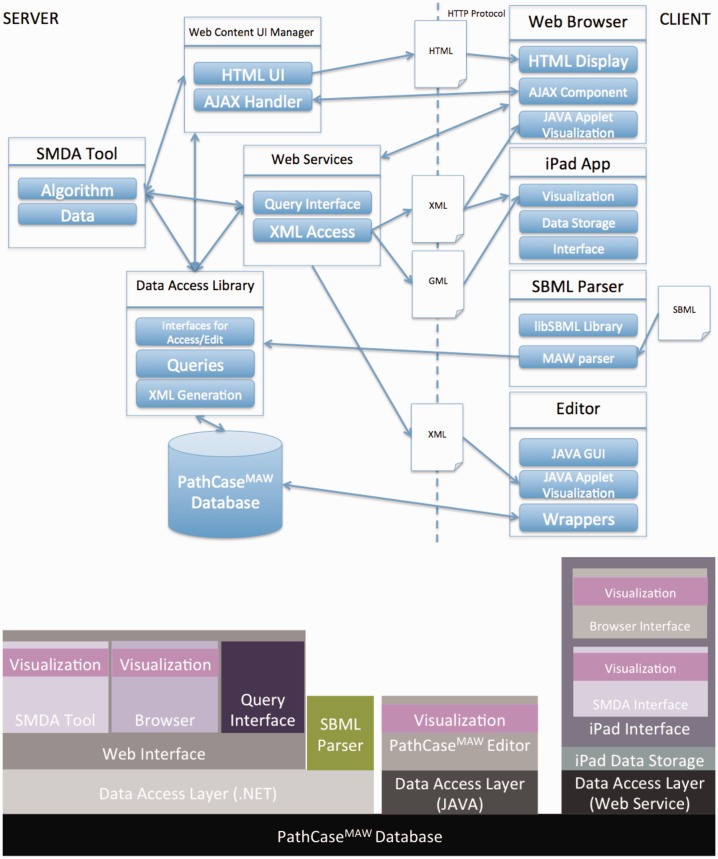
PathCase-MAW software architecture. Top: communication among components. Bottom: hierarchy among components.

Figure 1 illustrates the communication among the main components of the PathCase^MAW^ system (top), and shows the software architecture of the system (bottom). Details regarding the system are given in individual subsystem sections.

## Data access interfaces

In this section, we present the interfaces provided by PathCase^MAW^ system to browse, query, visualize and analyze the data. There are two main ways to access the data: (i) a Web browser (e.g. Google Chrome) and (ii) iPad App of PathCase^MAW^. Next, we explain the capabilities provided in each type of interface.

### Web browser

PathCase^MAW^ is an online system that can be accessed through an Internet browser. The backend is implemented in C#, using .NET framework. The Web site is hosted on a machine running Windows server 2008. We use Internet Information Services (IIS) 7.0 as our Web server. It has been tested on four major Web browsers with the recent versions of Microsoft Internet Explorer, Mozilla Firefox, Google Chrome and Apple Safari. The Web interface includes four applications, namely, (i) browser, (ii) visualization interface, (iii) querying interface and (iv) SMDA tool.

This interface lets the user to view the data stored in the database from various different starting points. For instance, the user can start from a metabolite to list related reactions, to find the pathways this metabolite plays a role in or one can start from a pathway to find related reactions and the compartments where each of these reactions takes place.

The browser interface has two parts: the navigation bar on the left hand side, and the main frame on the right hand side that shows details of the selected browser item. The left hand side is called the ‘Browser’ page, and the right hand page is called the ‘Details’ page.

**Figure 2.**
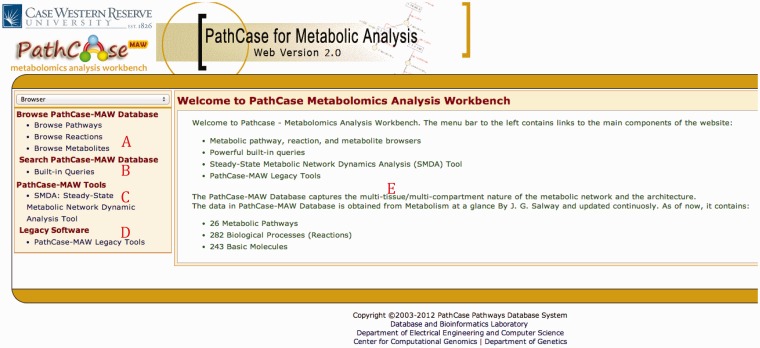
PathCase-MAW homepage. (**A**) Browser links to pathways/reactions/metabolites stored in the database. (**B**) Link to the built-in queries. (**C**) Link to the SMDA tool. (**D**) Link to legacy tools. (**E**) Details page reporting summary statistics about the database.

Figure 2 shows the home page of PathCase^MAW^. The main browser page has three subsections. Part A redirects the user to browse the data starting from (i) pathways, (ii) reactions and (iii) metabolites stored in the database. Part B links to the query interface, Part C links to the SMDA tool and Part D links to legacy tools developed by our team. Part E shows the mainframe where contents are loaded after clicking a link. The browser page has the following flexible hierarchy that enables user to effectively browse related entities of the network.

1. Pathways → reactions in the selected pathway → compartments the selected reaction takes place → metabolites associated with the selected reaction in the selected compartment.
2. Reactions → compartments the selected reaction takes place and pathways this reaction is associated with → (selecting a compartment) metabolites associated with the selected reaction in the selected compartment.
3. Metabolites → pathways the selected metabolite is associated with → compartments the selected pathway takes place in → reactions of the selected pathway that exist in selected compartment.

Part B opens up the query interface, and Part C links to the implemented tools (currently only SMDA tool). Part D links the user to our legacy tools, namely, the OMA tool (13) and MQL (14), which are no longer maintained.

Part E shows the details page where information about the selected browser item is loaded as separate collapsible panels. In the home page, statistics about the database is shown along with a quick introduction. Users are able to click on any entity on the browser page (as described above), to open up the details page. The following information is displayed per entity:

- **Pathway**: Panels for (i) loading the applet that visualizes the pathway, (ii) listing reactions taking place in the pathway, (iii) listing connected pathways in the complete metabolic network and finally (iv) listing all available pathway-related built-in queries.
- **Reaction**: Panels for (i) listing involved metabolites, (ii) listing related pathways and finally (iii) listing all available reaction-related built-in queries.
- **Metabolite**: Panels for (i) listing related pathways, (ii) listing synonyms stored in the database for this metabolite (e.g. Fructose 1,6 bisphosphate; F16P; F1,6P) and finally (iii) listing all available metabolite-related built-in queries.
- **Compartment**: A panel to list all reactions that take place in this compartment, and a panel to list all pathways that take place in the selected compartment.

**Figure 3.**
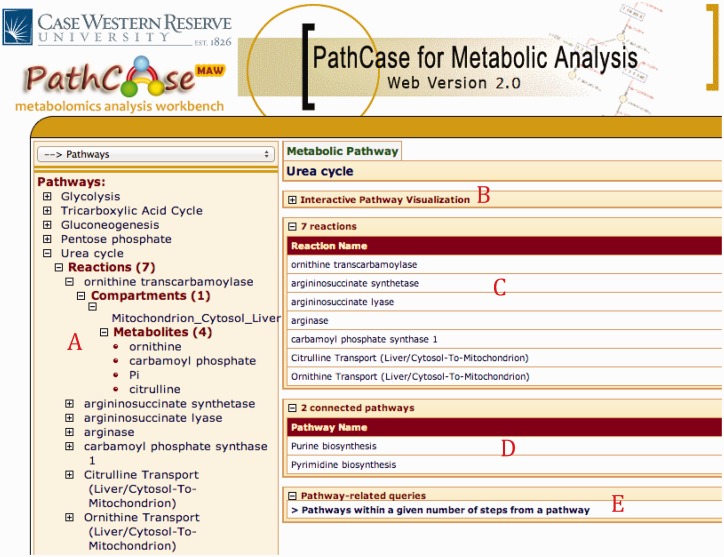
PathCase-MAW Browser and Detail pages for the pathway urea cycle

Figure 3 shows browser and detail pages for ‘Urea Cycle’ pathway. Clicking ‘Urea Cycle’ on the browser page loads up the collapsible panels to the details page in the collapsed form. Part A shows the hierarchy underneath the pathway. There are seven reactions in the pathway; selecting ‘ornithine transcarbamolyse’ shows that it only takes place in mitochondrion in cytosol of a liver cell. Underneath mitochondrion, we find all four metabolite pools that are associated with ‘ornithine transcarbamolyse’.

**Figure 4.**
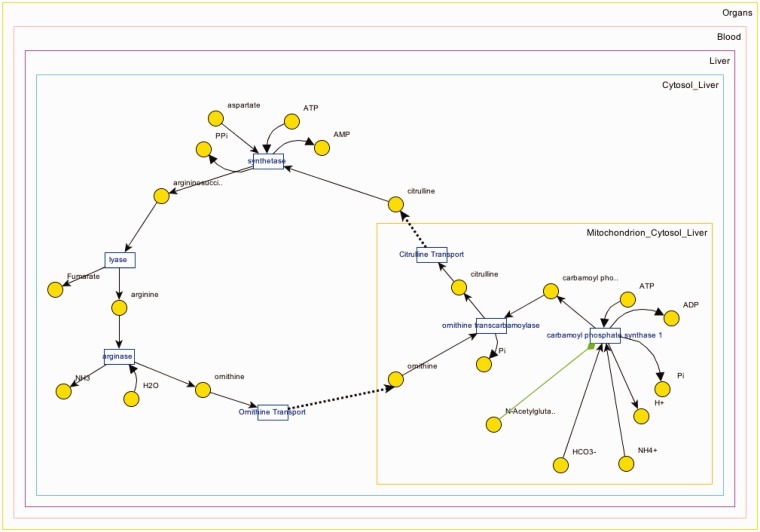
Visualization of the urea cycle pathway.

Panel B downloads the applet once clicked (only at the first usage) and loads up the XML data to visualize the pathway. A screenshot for the visualization of ‘Urea Cycle’ is shown in Figure 4, and details about the visualization interface are given in ‘Web/editor visualization’ Section. Panel C (in Figure 3) shows the reactions associated with ‘Urea Cycle’, and clicking each one of them would take the user to the details page regarding that reaction. Panel D shows there are two connected pathways to ‘Urea Cycle’ in the database through shared metabolite pool(s): ‘Purine Biosynthesis’ and ‘Pyrimidine Biosynthesis’. Finally, Panel E provides a link to a built-in query for finding pathways within a number of steps away from ‘Urea Cycle’.

The details page accesses the data using AJAX calls. Therefore, once an item is clicked, the collapsible panels are loaded. Collapsible panels make calls to the server after they are displayed, so the data are accessed asynchronously. If a collapsible panel is clicked, while the data are still being fetched, user is informed that it is being loaded. This brings a huge advantage in terms of user experience, when pathways with a large number of reactions and metabolites are accessed. In the synchronous case, users would have to wait for all panels to load before they see the page. Users may also interact with the loaded panels although some panels are still loading data in the background.

### Web-based SMDA tool

#### SMDA algorithm

SMDA (11, 12) uses an algorithmic approach to analyze metabolomics data in terms of the dynamic behavior of the metabolic network. Given a set of metabolite measurements, it identifies the metabolic activity over the network that lead to the observed concentration changes of given metabolite pools. The input data consist of one or more pathways; zero or more reactions, which constitute the selected metabolic subnetwork to be analyzed. Also, measured concentrations of specific metabolites within the selected metabolic subnetwork are provided as input. The observed concentrations of metabolites are compared against the normal/abnormal values of those metabolites reported in HMDB (15), and assigned one of the following labels: unavailable, available, accumulated and severely accumulated. Based on the biochemical principles and the input metabolite levels captured by the algorithm, SMDA algorithm determines whether a reaction is active at steady state. The algorithm produces possibly several scenarios where each scenario labels each and every reaction in the network as active or inactive. SMDA has been shown as an effective method to analyze subnetwork activation scenarios for cystic fibrosis research (11).

SMDA is both a constraint- and rule-based approach. It is constraint-based (16–18), as the search space is constrained by some biochemistry principles (19). It is rule-based in that its reasoning uses a number of basic biochemistry-based rules to capture the underlying metabolic biochemistry. In other words, SMDA approach specifies a complete condition- and rule-based model of the metabolic network behavior via the use of metabolite pool label identifiers, the three-valued logic to specify metabolite pool label conditions and the Activation Condition Sets for reactions as well as transport processes.

The SMDA algorithm runs in an iteration of two phases, namely, ‘Expansion and Merge’, and lasts until all reactions and metabolite pools in the network are assigned a status. Expansion phase starts from the labeled metabolite pools (observations from a given metabolomics measurement), which are flow graphs with single metabolite pool. Then, expanded flow graph(s) are generated by adding neighboring reactions and metabolite pools to the original flow graph. SMDA generates all possible combinations of label assignments to those neighboring pools and reactions. This process continues until all reactions and metabolite pools are assigned a label.

SMDA returns only two flux values for a reaction, namely, 0 (‘inactive’) and 1 (‘active’). The query output space of the SMDA tool is large. However, (i) larger numbers of observations exponentially reduce the output size, and (ii) exploratory search and browsing of the query output space allows users to mine and search for what they are looking for.

Advantages of SMDA include its ease of use and simplicity; it is designed as a ‘first-step’ and ‘online’ tool for wet-lab researchers (i) to evaluate their hypotheses about observed measurements, and (ii) to be used for ‘what if’ types of questions (i.e. knowledge discovery). SMDA technique and its computational performance limits are evaluated using a mammalian metabolic network database (11).

SMDA’s approach of evaluating the activation/inactivation scenarios of the metabolic network at steady state is related to metabolic network analysis techniques, such as MCA (1), FBA (2), metabolic flux analysis (20) and, finally, metabolic pathway analysis (elementary flux modes and extreme pathways) (21). MCA and FBA solve a set of under-constrained differential equations; in comparison, the SMDA approach can be considered as a constraint- and rule-based knowledge discovery approach within a given metabolic network database.

#### SMDA tool

**Figure 5.**
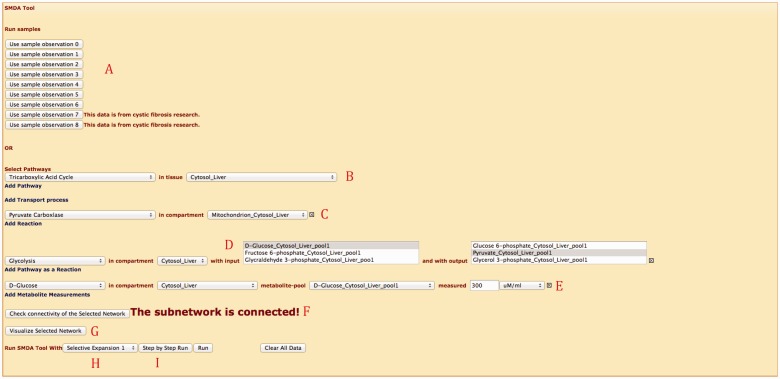
Web interface for SMDA tool.

SMDA tool implements the SMDA algorithm on server side and can be accessed via the Web interface. The tool is implemented using C# language in .NET environment. Next, we introduce the capabilities of the Web interface for the SMDA tool over an example, shown in Figure 5.

First, there are two options to input data. (i) Users can pick one of the eight provided sample input data shown in Part A. Clicking the button loads up the subnetwork selected and corresponding observations. (ii) Users can input data manually. For the second case, the user first needs to select the metabolic subnetwork of interest. In Part B, the user picks a pathway and a compartment (e.g. organ) that the pathway is taking place in (note that a pathway can exist in more than one compartment). In this example, ‘TCA Cycle’ in liver cell is selected. Next, in Part C, users select stand-alone reactions (which are not part of a pathway). They can select transport reactions or regular reactions. Transport reactions are unique so they do not require input of compartment, whereas regular reactions require the compartment to be specified. Users are also able to simplify pathways into a single reaction as done in many works in the literature. In this example, in Part D, ‘Glycolysis’ pathway is added as a single reaction that has ‘D-Glucose’ as input and ‘Pyruvate’ as output again in liver cell. Part E lets the user to pick metabolite pools and specify the concentrations manually. In this example, the only observation is ‘D-Glucose’ in the cytosol of liver cell. Finally, Part F concludes data input part by inputting observed enzyme levels for reactions. In Figure 5, ‘Citrate Synthase’ is observed as ‘Unavailable’. This observation deactivates the corresponding reaction(s).

After inputting data, users can check whether the selected subnetwork is connected and can visualize their network, using the buttons on Part G. Finally, the user can run the algorithm till it completes, or he/she can see how the algorithm proceeds (e.g. assigns labels to the next reaction set, and eliminate options) step-by-step by selecting one of the buttons shown on Part I. Part H lets the user to pick one of the iteration algorithms as explained in (11). SMDA tool is also equipped with AJAX calls. For instance, selecting a pathway opens up the compartment selection combo box, which loads only the compartments this pathway exists in.

The results are passed from the server back to the Web client in XML format and directly visualized. SMDA Visualization uses the same infrastructure used to visualize pathways. The only differences are (i) there are possibly more than one resulting graphs, each representing a possible scenario, and (ii) thin edges represent inactive reactions and thick edges represent active reactions.

### Web querying interface

PathCase^MAW^ currently has five built-in queries to enable users to find related pathways, reactions and metabolites with respect to another entity. The currently available queries are as follows:

1. *Pathways within a given number of steps from a pathway*: This query searches for pathways that are *n* reactions away from a given pathway.
2. *Reactions within a given number of steps from a reaction in a pathway*: This query searches for reactions that are *n* reactions away from a given reaction in a pathway.
3. *Reactions within a given number of steps from a reaction in the metabolic network*: This query searches for reactions that are *n* reactions away from a given reaction in the network.
4. *Reactions involving a metabolite in a pathway*: This query searches for reactions that involve a given metabolite in a specified role in a particular pathway.
5. *Metabolites within a given number of steps from a metabolite in the metabolic network*: This query searches for metabolites that are *n* reactions away from a given metabolite in the network.

The system is equipped with AJAX calls that prunes out irrelevant selections without reloading the page. For example, for the second query, the user is asked to pick a pathway, then a reaction. Once the user picks the pathway, the next combo box to pick the reaction in that pathway is loaded with only the reactions in that pathway, and the rest is discarded. This prevents the user from selecting illegal items (e.g. choosing a reaction not existing in the already selected pathway) and contributes to a positive user experience.

### Web/editor visualization interface

**Figure 6.**
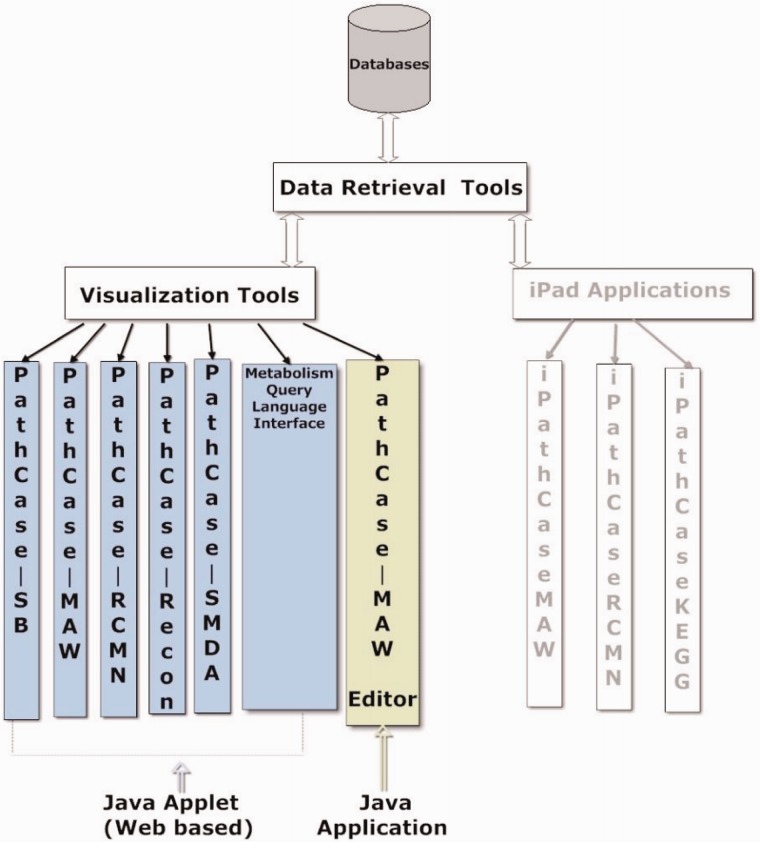
Visualization tools and applications.

PathCase visualization tools visualize metabolic data, relationships in the data, as well as analysis results of the data via multiple variations of a JAVA applet. The visualization tools are components of many PathCase Systems, as shown in Figure 6. In this section, we present the visualization tool in the PathCase^MAW^ system, namely, the PathCase^MAW^ visualization interface.

PathCase^MAW^ visualization interface is responsible for displaying interactive and user-friendly depictions of metabolic (sub)networks (e.g. pathways or a collection of pathways and reactions). It is used in three subsystems of PathCase^MAW^:

1. within a collapsible panel for pathway visualization on details page of a pathway,
2. as a tab for pathway visualization in the data editor and
3. in the SMDA tool, as a separate window for visualizing the selected subnetwork (any connected set of pathways and reactions) and visualizing the resulting flow graphs.

In the first two cases, the applet displays only the selected pathway. It uses the JAVA applet (based on yEd Graph Editor library) that runs on the client machine, and with no server-side intervention or communication other than an XML document exchange, through a Web service. This allows PathCase^MAW^ to scale for a large number of users. It is run within an Internet browser. The visualization interface seamlessly downloads the applet itself only at the time of the first use, and comes embedded into the corresponding pathway pages of the SMDA tool interface. This architecture has two advantages.

- The visualization does not require a separate installation effort (a manageability convenience for users) and
- the architecture provides a platform-independent access regardless of the operating system or browser that is run on the user machine. For the editor, it comes embedded within a jar file, and displays all selected pathways and reactions.

The visualization in PathCase^MAW^ and PathCase^MAW^ editor has the capability of visualizing a pathway in multiple tissues (if it exists in multiple tissues) side by side.

The interactive graph displays metabolites (circles), reactions (small rectangles), compartments (rectangles that contain metabolites and reactions and subcompartments) and the relations between them (as lines) in a metabolic (sub)network. The directions of the lines set producer/ consumer relation for a reaction (bidirectional lines indicate reversible reactions). Red/green line indicates inhibitor/activator relationships, respectively. Interface captures the compartment hierarchy. Metabolite pools and reactions are shown in the corresponding compartments.

Users are able to interact with the visualization; they can (i) move around all visualized items, (ii) zoom in/out in different ways (e.g. fit-to-screen, magnifier, etc.) and (iii) save the modified layout so that it appears the same layout on the next load for that pathway/subnetwork (password protected for data owner).

**Figure 7.**
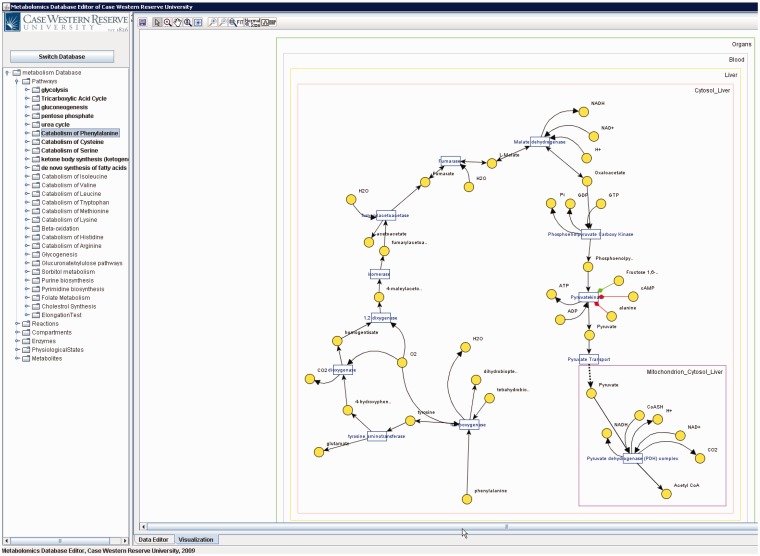
Catabolism of phenylalanine pathway in PathCase-MAW editor.

Metabolites that participate in many reactions (e.g. H_2_O, NAD, ATP) have high connectivity and tend to result in unclear visualizations. Such metabolites are called ‘common (also known as currency) metabolites’. Unlike all metabolite pools, which are displayed once, these metabolites are displayed per participated reaction to provide simpler visualization (see Figure 7).

Based on the yEd framework, the applet provides various detailed functionalities. Applet provides tooltips for the user, that is, when the cursor is moved over an item, the corresponding entity name (e.g. reaction or metabolite name) is displayed in a little box that pops up and disappears when cursor is moved out. This lets the applet to keep truncated forms of the long names and keep the image tidy, while letting the user see the names of interest in advance. Another useful functionality is the map, which is crucial for large pathways/subnetworks. Picking this option opens up a small and zoomed-out bird’s-eye snapshot of the complete visualization. This way, the user is able to navigate through the visualization without a need to zoom-out and then zoom-in again.

### iPad interfaces

iPad has been a revolutionizing mobile device with a multi-touch interface that has sold 9.25 million only in the fiscal 2011 third quarter (22). Other tablet companies have followed, and it has been a widespread technology. More widespread the tablets are, more attention they attract for educational purposes. For instance, Apple has launched an effort to revolutionize textbooks (23), and countries (such as Turkey) distribute tablets to students (24) to use as part of their classes. Our goal in building an iPad app for PathCase^MAW^ is to provide educational tools for life scientists in biochemistry teaching and research. Users are able to have nice visual depictions of pathways and run the SMDA tool using the iPad interface. The system is written in Objective-C language using Cocoa framework. Although the system works on iPad 1, currently, the system is optimized for efficient use on iPad 2 or higher (which is called iPad from now on). The system has three main interfaces: browsing, visualization and SMDA tool interfaces. Next we give details of each interface.

#### iPad browser interface

**Figure 8.**
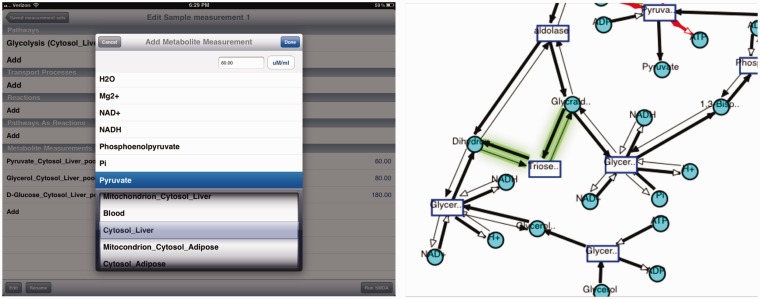
iPad visualization interface. Top: Visualization of cholesterol synthesis pathway on iPad is shown along with the legend. Bottom: running SMDA on iPad for glycolysis pathway.

The browser of the application consists of only pathway browsing that acts as a link to the visualization of a pathway. At the current stage, reaction, metabolite and compartment browsing is not supported. Pathway browser can be accessed through the home page, where pathways are listed on the right frame, or from within a visualization of any pathway by tapping the ‘Pathway’ button in the toolbar as seen on the top right of Figure 8 (top).

#### iPad visualization interface

The visualization interface used for iPad looks similar to the Web interface’s visualization; however, the implementation is different. The application uses Core Animation Framework to render objects. The data are obtained in the form of an XML + GML (Graph Markup Language) document via Web services. Because of the hierarchical and interconnected nature of the data model, it is not practical to dynamically invoke the Web services at the time their data are needed. Instead, an internal representation is updated in bulk at the user’s request. When the user taps on the ‘Update Local Data’ button in the SMDA input view, a series of asynchronously executed Objective-C blocks are invoked. This design choice cuts down on the data quote usage of the device and provides fast response time for the user.

#### iPad SMDA tool interface

SMDA tool interface uses the same server-side component that implements the SMDA algorithm. It provides a similar interface as the Web interface, which lets the user to pick the subnetwork and the measurements for metabolites. Results are visualized in the same manner (thick edges represent active reactions and thin edges represent inactive reactions). As stated before, there are possibly more than one resulting visualizations. The iPad visualization interface implements a nice feature to mark the differences between the current image and the previous one.

Figure 8 displays a run of the SMDA tool on ‘Glycolysis’ pathway. On the left, the input is shown, and on the right, one of the four results is displayed. The glowing green edge shows the difference between the previous possible scenario and this one. Users can switch between results by a one finger swipe to right and left.

## Data input tools

In this section, we describe two tools used to manage the data stored in PathCase^MAW^ database.

### PathCase^MAW^ editor

Entering metabolomics data manually into a relational database is a complicated and error-prone process. A user in our context is the data owner (e.g. metabolic network) who would like to use our system to analyze the data. A user obtains components of PathCase^MAW^ (e.g. editor, Web interface) and populates its own metabolic network database. Consider a user entering a new reaction (with a new substrate and a new product). First, she needs to enter two new metabolites into the ‘Metabolite’ table and then two new entries into the ‘MetabolitePool’ table keeping the IDs of the metabolites in mind. Next, she needs to add the reaction into the ‘Reaction’ table. Finally, she needs to add two entries into ‘ReactionMetabolite’ table coupling the IDs of the metabolite pools and the reaction along with the role IDs of the metabolite pools. Thus, even entering a single reaction is a complicated and an error-prone task. To enable users to manage the data easily, we have developed an easy-to-use editor (25) that houses tools for (i) populating, (ii) editing and (iii) visualizing the data stored in PathCase^MAW^ database. The editor, built as a JAVA application (and therefore, platform independent), provides a user-friendly tool to access and modify the metabolism database, as well as to review the modifications visually. It (i) checks data dependency internally to prevent data integrity violations, and (ii) ensures data correctness during data insertion and editing to help users input valid data, and also improve data insertion efficiency. Via a user-friendly graphical user interface, the editor allows (i) editing of pathways, reactions, compartments, enzymes, metabolites and metabolite pools, physiological states and (ii) visualization of pathways either within the main editor window or as a separate stand-alone full-screen window. Below is a more detailed summary of the editor. For more details, please see Han’s thesis (25).

PathCase^MAW^ editor has the following functionalities/advantages:

1. The system hides from users the internal object IDs. All relationships are shown via object names in the GUI, and the user cannot modify object’s ID directly; this effectively eliminates much of the data inconsistency possibilities.
2. PathCase^MAW^ editor is designed to prevent entering illegal data by providing a drop-down box for a field with fixed set of allowed values, rather than providing a free-text box. In cases where the first measure fails, it explicitly checks the data against certain constraints.
3. Users can insert new data in two ways: (i) they can insert a new entity (i.e. from scratch) and (ii) for some object types (i.e. compartment), they can copy an existing object, modify and insert it into the database as a new record.
4. To input data efficiently, PathCase^MAW^ editor provides an autocomplete feature for text fields. Given the complexity of the biological terminology, this feature saves unnecessary keystrokes and prevents illegal data insertion to the database.
5. Changes made on the data are not reflected to the database right away. Users need to approve their changes by ‘saving’ their work to avoid updating the database by mistake.
6. The integrated visualization applet enables user to see results of the update on the fly, which acts as an efficient visual debugger.

**Figure 9.**
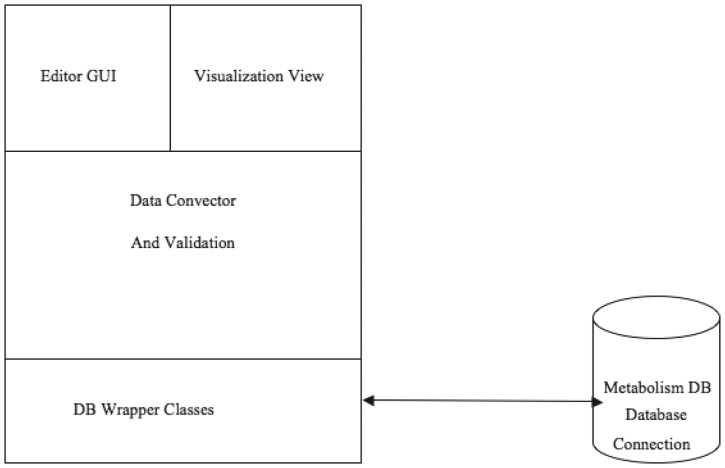
PathCase-MAW editor architecture.

Figure 9 illustrates the software architecture of ‘PathCase^MAW^ Editor’. The lowest layer, ‘database wrapper classes’, contains a set of wrapper classes for the Java Database Connection. This layer (i) initializes and maintains the database connection, and (ii) via SQL queries, provides an easy-to-use interface for select, insert, update and delete queries to the upper level. The middle layer, ‘Mediation Layer’, performs data conversion and validation. This layer contains two abstract classes, namely, ‘BaseEntity’ and ‘BaseRelation’ through which common database operations (e.g. creating new entities, deleting entities, selecting by common criteria, etc.) are implemented. The actual (‘real’) entity’s class is derived from BaseEntity, and the real relationship class is derived from BaseRelation. Each real entity class is mapped to one object (e.g. pathway, metabolite, etc.) table in database, and each real relationship class is mapped to one relationship (e.g. PathwayReaction, ReactionMetabolite) table in database. The upper layer, the ‘GUI layer’, uses the ‘java reflection’ technique to dynamically retrieve real entity/relationship classes, so that the name of the real database class is transparent to the upper layer. This means that, when the database schema is changed (e.g. to add new object type), the upper layer code (e.g. the editor GUI) stays the same, providing flexibility.

**Figure 10.**
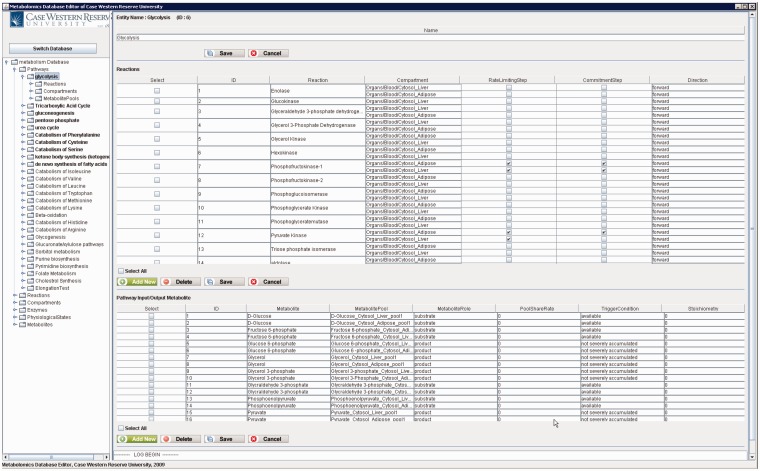
PathCase-MAW editor main frame.

Figure 10 shows the main frame of ‘PathCase^MAW^ Editor’, which includes two subpanels. The left panel contains an ‘Object Tree’ that presents the object hierarchy with the root ‘Pathway’. The object hierarchy lists reactions, compartments and metabolite pools, which are related to the given pathway. The maximum depth of the tree is 3 (levels of objects). The right panel includes two tabs: ‘Data Editor’ tab for data viewing and editing and ‘Visualization’ tab for visualization view. In the data editor Panel, the top part shows the current object’s name, ID and other information. The middle part lists tables that contain relationships to the currently edited object. The operation results (e.g. how many rows deleted from the database) are placed at the bottom part.

The third layer is the GUI layer with two parts: one is the editor GUI that provides data insertion, editing and viewing operations, and the other one is the visualization viewer.

**Figure 11.**
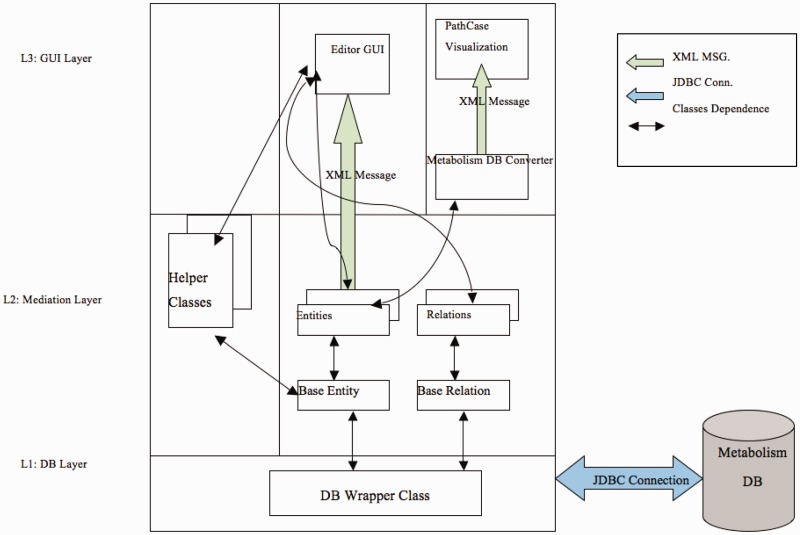
Data workflow within software architecture.

Figure 11 presents the data workflow within the software architecture. Communication between the editor GUI and database classes is via XML documents. For the visualization viewer, an XML document capturing the present pathway in the metabolism database is parsed and passed to the visualization viewer.

**Figure 12.**
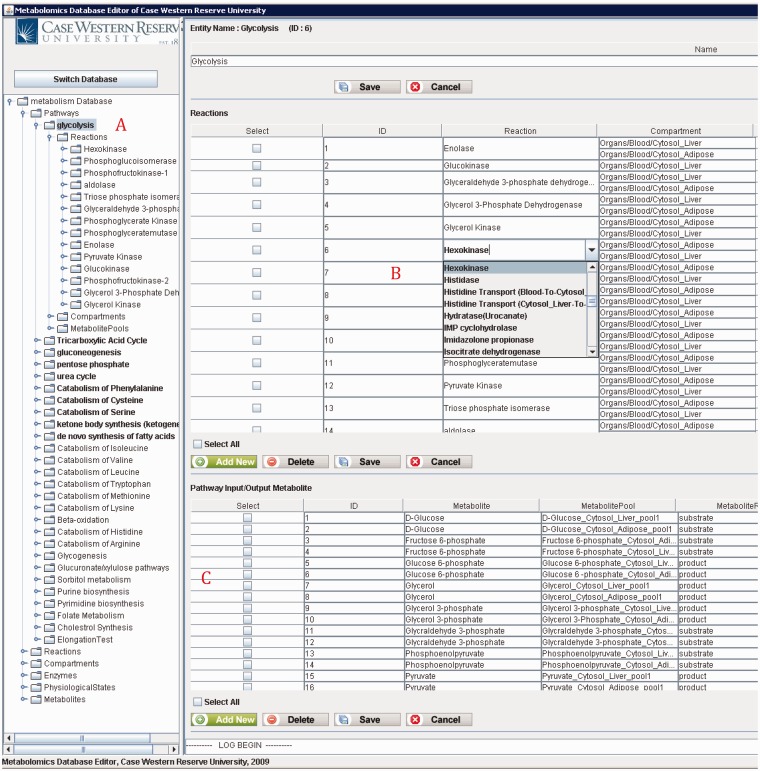
PathCase-MAW editor showing data for glycolysis pathway.

Figure 12 shows a screenshot of the editor mainframe. Part A on the left hand side shows the navigation hierarchy for each entity (e.g. pathways, reactions, compartments). In this example, the pathway ‘Glycolysis’ is chosen, and the reactions belonging to the pathway are shown. Similarly, one can go one more level down to see the metabolite pools associated with these reactions. The same logic applies to other nodes shown on the tree. For instance, toggling ‘Metabolites’ group would list all metabolites, and going down one more step would show the pathways, in which this metabolite participates. Part B on the top right-hand side shows the reactions associated with ‘Glycolysis’ pathway in a grid form. In Figure 12, reaction ‘Hexokinase’ is clicked, which opens up a drop-down box listing all reactions available in the database. The user can replace ‘Hexokinase’ using this drop-down box. This feature prevents users from entering illegal entries to the database. Part C shows the input/output metabolites for ‘Glycolysis’. Right below Part C, there are two tabs. Selecting ‘Visualization’ tab shows the visualization of ‘Glycolysis’ pathway for liver and adipose tissues. The visualization applet is shared by the Web interface, and details about the visualization interface are discussed in ‘Web/editor visualization interface’ Section.

Although we have presented a few examples of updating pathways, reactions or metabolites, the editor has a large number of user-friendly features (25):

- Compartment tree support,
- Automatic metabolite pool creation,
- Compartment duplication for reuse,
- Autocomplete feature for object fields, and
- Selection lists for enumerated fields.

The editor also provides different convenient ways for users to interact with the database (25).

### PathCase^MAW^ SBML parser

SBML (26) is one of the standards to create and distribute systems biology models. Although it has been used to describe many kinds of systems, in the context of PathCase^MAW^, we are dealing with metabolic networks that have been described in SBML format. PathCase^MAW^ SBML parser is a descendent of PathCase^SB^ SBML parser (27), a subsystem of PathCase^SB^ project. PathCase^SB^ (28–30) is a system that stores kinetic models of pathways provided by BioModels Database (31), and offers extensive functionalities over the model data by linking multiple resources. PathCase^SB^ SBML parser is used to parse SBML, and to populate the PathCase^SB^ database (29). Schemas and functionalities of PathCase^SB^ and PathCase^MAW^ databases are different. PathCase^SB^ database focuses on integrating multiple resources, whereas PathCase^MAW^ database is intended to effectively store and analyze metabolic networks. PathCase^SB^ SBML parser’s data access layer has been completely changed to suit PathCase^MAW^ database schema, and the XML processing component is modified to handle differences between description of kinetic models for pathways and description of metabolic networks. The challenges and problems with SBML format misuse have been discussed in ‘Results and Discussion’ section in detail.

The parser is written in C# language using .NET framework. It relies on the libSBML library (currently Version 1 of Level 3) (32), which is a public library that provides a framework for parser to access elements of the SBML model. As the model is parsed, the database is accessed using the Data Access Library that contains the object-oriented wrapper classes and their functions. Wrapper classes are shared by the browser code, so any change made on the database schema can be handled easily by changing the related wrapper classes. These wrapper classes are different than editor’s wrappers, as they are implemented in JAVA language. System parses the file stored in a given directory and updates the database accordingly.

## Results and discussion

All presented subsystems of PathCase^MAW^ have been implemented and released to the research and community. Currently, the system runs on a manually created generic mammalian database that consists of 26 pathways, 282 reactions and 243 metabolites. We have also parsed and released three genome-scale reconstructed metabolic networks (two for *Mus musculus* and one for *Trypanosoma* *cruzi*) in the sister PathCase^RCMN^ site. Next, we provide a comparison of the existing systems with PathCase^MAW^ and then the challenges/shortcomings of the current system.

### Comparison of PathCase^MAW^ with existing systems

KEGG (33–36) has been a major source of metabolic pathways, which provides an application programming interface and data download options. Unlike PathCase^MAW^, KEGG (i) provides limited visualization and browsing capabilities, (ii) does not capture compartment information for metabolites, (iii) provides limited functionality over the data and (iv) ignores metabolic regulation, such as covalent activation/inhibition or metabolite ratios (e.g. high NAD/NADH ratio activates alpha-ketoglutarate dehydrogenase).

PathCase^SB^ and PathCase^KEGG^ (37) have been released to provide additional functionalities such as querying and interactively visualizing pathway data. PathCase^KEGG^ hosts KEGG data in its own schema and provides similar browsing and visualizing capabilities as PathCase^MAW^ system. PathCase^SB^ stores kinetic models of pathways provided by Biomodels Database (31). It integrates KEGG data with the stored models, and enables users to simulate and compose models. However, neither of these systems have metabolomics analysis goals or tools. They only work on data provided by well-known third parties (KEGG and Biomodels Database). Conversely, our goal is to provide a system that enables users to work on their own data to use the functionality provided. Finally, among the three above-mentioned systems, only PathCase^KEGG^ has a mobile interface (38) (iPad application).

BioCyc (39) is another major pathway/genome database, which hosts numerous different organism databases at various curation levels. They provide tools like PathoLogic (40) to predict and reconstruct metabolic networks directly from the corresponding genomes, and SRI’ss pathway tools to adapt and curate networks. For an organism, the database contains the following information: genome, gene products, metabolic network, regulatory network and the transporter complement of the organism. Although the data are comprehensive (e.g. PathCase^MAW^ is only dealing with metabolic networks), browsing and visualization capabilities are limited on their Web site. BioCyc provides a SVG-based, static visualization of the complete metabolic network, and links to related sources per item, but there is no other interaction, unlike the PathCase^MAW^ visualization interface. ‘Pathway Tools’ (41) provide such features; however, it is an offline system and can be compared with the PathCase^MAW^ editor. Computational tools provided by pathway tools focus on prediction of metabolic features, such as pathways, choke points or operons for metabolic networks. On the other hand, SMDA tool is concerned with analyzing metabolic profiles and predicting metabolic activity on metabolic networks. They also provide a FBA tool, but only in the desktop version. BioCyc does have an iPhone application for only EcoCyc (42), and there is no application for iPad. In short, BioCyc metabolic network creation from the genome is a big (and, as they also state, difficult) and different task than PathCase^MAW^’s task, which deals with already created networks such as those from already reconstructed genome-scale networks. PathCase^MAW^ clearly provides a much smaller subset of the information provided by BioCyc; however, for metabolic networks, it provides a user-friendly, manageable and online system with nice analysis, browsing and visualizing capabilities.

Reactome (43–46) is a pathway database curated and updated by life scientists. Although the visualization is static (e.g. no moving around of items), it provides beautiful images of selected pathways with zoom in/out capabilities and links to related resources (based on Systems Biology Graphical Notation). Reactome provides tools such as pathway analysis (e.g. overrepresentation analysis, comparing pathways of species). They also provide a built-in query interface similar to PathCase^MAW^. There is no mobile interface provided. Reactome’s goal is to have an all-inclusive one-stop-shop type of curated metabolic pathway database, whereas PathCase^MAW^’s goal is to provide tools and schema for researchers to move their own data to PathCase^MAW^ schema to have the access, query, browsing and analysis interfaces available.

MEMOSys (47) is a system that provides a platform for researchers to collaborate and create reconstructed metabolic networks. The system has a version control mechanism to keep track of the history of the models. The networks that are already created are stored in a repository. They can be browsed, compared and exported via the user-friendly Web interface. They provide PDF-based maps for some models. However, the system does not offer metabolomics analysis tools, query interface, mobile interface or interactive visualizations.

In summary, PathCase^MAW^ is a unique system with its goal and capabilities. It has similarities and differences, compared with the existing systems. However, it is not a stand-alone advancement over an existing system.

### Challenges and shortcomings

One of the biggest challenges of the PathCase^MAW^ system is with parsing reconstructed metabolic networks. And, the reason is the misuse of the SBML format for creating networks by researchers. For instance, Edinburgh metabolic network for humans (48) does not specify ‘compartment’ attribute for species, as they do not distinguish between compartments, and assume all information is in the same single compartment, ‘cell’. Similarly, many models include information (e.g. associated pathway/subsystem for a reaction) in the tag, which is simply unstructured plain text from the parser’s point of view, and creates parsing problems for all SBML parsers. These challenges are explained in detail in Alshalwi, 2011 (49). The second challenge is with the exponential complexity of the SMDA Tool, which is also explained in detail in Cakmak *et al.*, 2012 (11). For networks with high numbers of reactions and, in those cases, with a small number of metabolite observations, the SMDA system may have an unacceptably long response time, and may even run out of memory. That said, note that this is a problem shared by all constraint-based methods (16) with similar goals and assumptions.

## Conclusions and future work

PathCase^MAW^ is an online multi-tool system to effectively create, store, browse, query, visualize and analyze metabolic networks. The system provides an SBML parser to create networks from a SBML document, and a user-friendly interface that lets users to update the database. Stored information can be accessed online either through a Web interface or an iPad application. PathCase^MAW^ is a useful system to access and user-created reconstructed networks online for researchers. An analysis made online on the SMDA tool has been published (11) as a proof of concept.

As future work, our goal is to link the data stored in the database to outside sources such as KEGG for reaction references and HMDB for metabolite references, whenever the information is provided in the input model. Next, we plan to have a collective site that includes all of the released models for the use of research community.
